# Comprehensive methylation analysis of imprinting-associated differentially methylated regions in colorectal cancer

**DOI:** 10.1186/s13148-018-0578-9

**Published:** 2018-12-04

**Authors:** Hidenori Hidaka, Ken Higashimoto, Saori Aoki, Hiroyuki Mishima, Chisa Hayashida, Toshiyuki Maeda, Yasuo Koga, Hitomi Yatsuki, Keiichiro Joh, Hirokazu Noshiro, Ryuichi Iwakiri, Atsushi Kawaguchi, Koh-ichiro Yoshiura, Kazuma Fujimoto, Hidenobu Soejima

**Affiliations:** 1 Division of Molecular Genetics and Epigenetics, Department of Biomolecular Sciences, Faculty of Medicine, Saga University, Saga, Japan; 2 Department of Internal Medicine and Gastrointestinal Endoscopy, Faculty of Medicine, Saga University, Saga, Japan; 3 Department of Obstetrics and Gynecology, Faculty of Life Sciences, Kumamoto University, Kumamoto, Japan; 4 Department of Human Genetics, Nagasaki University Graduate School of Biomedical Sciences, Nagasaki, Japan; 5 Department of Pediatrics, Faculty of Medicine, Saga University, Saga, Japan; 6 Department of Surgery, Faculty of Medicine, Saga University, Saga, Japan; 7 Section of Clinical Cooperation System, Center for Comprehensive Community Medicine, Faculty of Medicine, Saga University, Saga, Japan

**Keywords:** Genomic imprinting, DNA methylation, iDMR, CIMP, *BRAF* mutation, *KRAS* mutation, LINE-1, *IGF2*-DMR0, *IGF2* LOI

## Abstract

**Background:**

Imprinted genes are regulated by DNA methylation at imprinting-associated differentially methylated regions (iDMRs). Abnormal expression of imprinted genes is implicated in imprinting disorders and tumors. In colorectal cancer (CRC), methylation and imprinting status of the *IGF2/H19* domain have been studied. However, no comprehensive methylation analysis of iDMRs in CRC has been reported. Furthermore, the relationship between iDMR methylation status and other methylation-related issues, such as CpG island methylator phenotype (CIMP) and long interspersed element-1 (LINE-1) methylation, remains unclear.

**Results:**

We analyzed the methylation status of 38 iDMRs in 106 CRC patients. We also investigated CIMP, LINE-1 methylation, *KRAS* and *BRAF* gene mutations, and loss of imprinting (LOI) of *IGF2*. We further examined the relationship between these factors and clinicopathological factors. The overall trend in iDMR methylation was towards hypermethylation, and iDMRs could be grouped into three categories: susceptible, resistant, and intermediate-to-aberrant methylation. The susceptible and resistant iDMRs consisted of all types of iDMR (gametic and somatic, maternally and paternally methylated). Hypermethylation of multiple iDMRs (HyMiD)-positive status was statistically associated with CIMP-positive status, but not associated with mutations in the *BRAF* and *KRAS* genes. HyMiD-positive status was inversely associated with LINE-1 methylation. Among four iDMRs within the *IGF2/H19* domain, *IGF2*-DMR0 hypomethylation occurred most frequently, but was not associated with *IGF2* LOI. Finally, we statistically calculated predictive prognostic scores based on aberrant methylation status of three iDMRs.

**Conclusion:**

In CRC tissues, some iDMRs were susceptible to hypermethylation independent of the type of iDMR and genomic sequence. Although HyMiD-positive status was associated with CIMP-positive status, this was independent of the *BRAF* and *KRAS* pathways, which are responsible for CIMP*.* Since *IGF2*-DMR0 hypomethylation and aberrant methylation of other iDMRs within the *IGF2/H19* domain were not associated with *IGF2* LOI, dysfunction of any of the molecular components related to imprinting regulation may be involved in *IGF2* LOI. The prognostic score calculated based on aberrant methylation of three iDMRs has potential clinical applications as a prognostic predictor in patients. Further study is required to understand the biological significance of, and mechanisms behind, aberrant methylation of iDMRs and *IGF2* LOI in CRCs.

**Electronic supplementary material:**

The online version of this article (10.1186/s13148-018-0578-9) contains supplementary material, which is available to authorized users.

## Background

Genomic imprinting is an epigenetic phenomenon resulting in differential expression of a subset of mammalian genes in a parent-specific manner. Generally, imprinted genes form clusters in certain chromosomal regions called imprinting domains. The expression of imprinted genes is regulated by epigenetic mechanisms, especially DNA methylation, at imprinting-associated differentially methylated regions (iDMRs) within these domains [1, 2]. iDMRs are characterized by DNA methylation on one of the two parental alleles. iDMRs are classified as maternally methylated and paternally methylated, as well as gametic or somatic. In gametic iDMRs, DNA methylation is acquired during gametogenesis and maintained from zygote to somatic cell. Most gametic iDMRs are present in imprinting control regions (ICRs), while somatic iDMRs are established during early embryogenesis after fertilization under the control of nearby ICRs [1]. Many imprinted genes play important roles in cell growth and differentiation, embryogenesis, fetal development, placental formation, and metabolism [3]. Loss of imprinting (LOI), or loss of monoallelic expression due to aberrant DNA methylation at iDMRs, is implicated in imprinting disorders such as Beckwith-Wiedemann syndrome, Angelman syndrome, and Prader-Willi syndrome [2, 4, 5].

Abnormal expression of imprinted genes is also implicated in the pathogenesis of cancers, because many imprinted genes regulate cell growth and differentiation [6]. *IGF2* LOI occurs in childhood tumors such as Wilms tumor, hepatoblastoma, and rhabdomyosarcoma, as well as in a majority of adult tumors, such as prostate, breast, lung, colorectal, and liver cancer [7]. *IGF2* LOI in Wilms tumor and hepatoblastoma is reportedly correlated with hypermethylation of *H19*-DMR, which is the ICR in the *IGF2/H19* domain [8, 9]. Disrupted expression of other imprinted genes has also been reported in various other cancers. For instance, in esophageal cancer cell lines, silencing of *CDKN1C* was correlated with hypomethylation of *Kv*DMR1, which is the ICR in the *KCNQ1OT1* domain [10]. Hypermethylation of *ARHI* and *PEG3* iDMRs, which are imprinted tumor suppressor genes, is associated with downregulation of these genes in ovarian cancer [11]. It has also been proposed that the expression and methylation status of several imprinted genes are associated with clinicopathological factors. For example, *IGF2*-DMR0 hypomethylation is associated with shorter survival in colorectal cancer (CRC) patients [12]. *ARHI* expression is associated with prolonged disease-free survival in epithelial ovarian cancer and pancreatic endocrine tumor patients [13, 14].

CRC is the third most common cancer globally in men and the second most common in women [15]. Among imprinted domains, the *IGF2/H19* domain has been well studied in CRC. It was reported that *IGF2* LOI was associated with *IGF2*-DMR0 hypomethylation, but not *H19*-DMR hypermethylation [16]. *IGF2* LOI in lymphocytes has been suggested as a potential risk predictor in CRC [17], while a different study contested the association between *IGF2*-DMR0 hypomethylation and *IGF2* LOI in CRC [18]. Additionally, the association was not detected in osteosarcoma, ovarian, or breast cancer [19–21]. The relationship between *IGF2* LOI and *IGF2*-DMR0 hypomethylation thus remains unclear. To date, no comprehensive methylation analysis of iDMRs in CRC has been carried out. In this study, we comprehensively analyzed the methylation status of 38 iDMRs in tumors and adjacent normal mucosae from 106 CRC patients. We also investigated CpG island methylator phenotype (CIMP) status, long interspersed nuclear element-1 (LINE-1) methylation, mutations in *BRAF* and *KRAS*, and allelic expression of *IGF2*. Finally, we examined the relationship between these genetic and epigenetic factors and clinicopathological factors in CRC.

## Results

### CRC patient clinical information

A total of 106 CRC patients were included in this study. Patients with metastases, preoperative chemotherapy, and inflammatory bowel disease were excluded. We analyzed paired frozen tumor tissues and adjacent normal mucosae. Patient clinical information is provided in Additional file 1: Table S1. Sub-division of the groups by site of tumor occurrence (right colon, left colon, or rectum) revealed no statistically significant differences in terms of age at diagnosis, sex, cancer stage, lymph node metastasis, or pathological type (Additional file 1: Table S1).

### Methylation status of 38 iDMRs

**Fig. 1:**
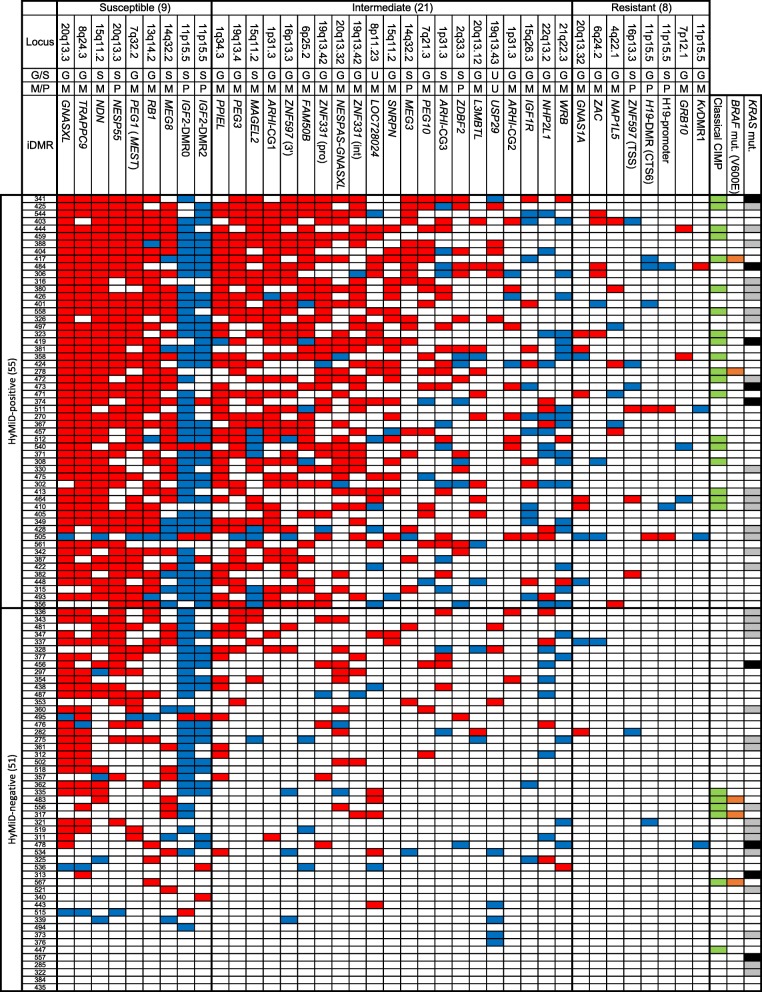
Methylation statuses of 38 iDMRs, CIMP status, and *BRAF* and *KRAS* mutations in 106 CRC patients. The three-digit number in the leftmost column indicates each patient’s ID. Hypermethylation and hypomethylation of iDMR are indicated by red and blue, respectively. CIMP-positive samples are indicated in green in the column on the right. The *BRAF* mutation (V600E) is indicated in orange. The *KRAS* (G13D) mutation and other *KRAS* mutations are indicated in black and gray, respectively. G, gametic iDMR; S, somatic iDMR; M, maternally methylated iDMR; P, paternally methylated iDMR; U, unidentified; *ZNF597* (TSS), transcription start site region of *ZNF597*; *ZNF597* (3′), 3′ region of *ZNF597*; *ZNF331* (pro), promoter region of *ZNF331*; *ZNF331* (int), intron of *ZNF331*. Definitions of aberrant methylation of iDMR and CIMP-positive are provided in the “Methods” section

**Fig. 2:**
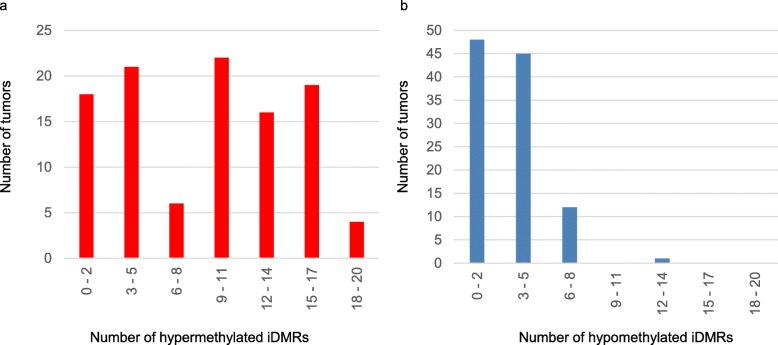
Frequency of iDMR aberrant methylation. **a** The distribution of hypermethylated iDMRs in tumors by number. **b** The distribution of hypomethylated iDMRs in tumors by number. The data indicate that iDMRs are more susceptible to hypermethylation than hypomethylation in CRC tissues

We performed a preliminary assessment of methylation status of 45 iDMRs, including iDMRs identified by Court et al. [22], in all adjacent normal mucosae by bisulfite pyrosequencing. We found that 37 iDMRs were differentially methylated with a high degree of precision (standard deviation < 10% of methylation). *IGF2*-DMR2, which was hypermethylated (87% methylation) in normal mucosae, was included because of its location within the *IGF2* gene. Therefore, we analyzed 38 iDMRs in this study. The methylation statuses of all iDMRs in tumor tissues were summarized into a qualitative chart (Fig. 1) (see the “Methods” section for the definition of aberrant methylation). The median number of hypermethylated iDMRs per tumor was 10 (ranging from 0 to 19), and of hypomethylated iDMRs was 3 (ranging from 0 to 12), indicating that hypermethylation of iDMRs is more likely in CRC tissues (Fig. 2a, b). In addition, we found that iDMRs could be grouped into three categories based on their susceptibility to aberrant methylation (Fig. 1). The first group was susceptible and consisted of nine iDMRs aberrantly methylated in half of all tumors or more (53 or more) (Fig. 1). Seven of these (*GNASXL*, *TRAPPC9*, *NDN*, *NESP55*, *PEG1*, *RB1*, and *MEG8*) were predominantly hypermethylated, although *IGF2*-DMR0 and *IGF2*-DMR2 were predominantly hypomethylated. The second group exhibited resistance to aberrant methylation and consisted of eight iDMRs (*GNAS1A*, *ZAC*, *NAP1L5*, *ZNF597* (TSS), *H19*-DMR, *H19*-promoter, *GRB10*, and *Kv*DMR1) that were aberrantly methylated in less than 10% of tumors (under 11) (Fig. 1). The last group was an intermediate group consisting of the remaining 21 iDMRs (Fig. 1). These findings indicate that susceptibility to aberrant methylation varies according to the individual iDMR and that certain iDMRs were highly susceptible to aberrant methylation while others were resistant. The susceptible group as well as the resistant group consisted of both gametic and somatic iDMRs as well as both maternally and paternally methylated iDMRs. In addition, we could not find a consensus sequence among the representative five hypermethylated iDMRs (*GNASXL*, *TRAPPC9*, *NDN*, *NESP55*, and *PEG1*). These results suggest that susceptibility to aberrant methylation does not depend on iDMR type or genomic sequence.

### Relationship between hypermethylation of multiple iDMRs (HyMiD) and CIMP

Because the median number of hypermethylated iDMRs per tumor detected in our study was 10, we defined tumors with hypermethylation in 10 or more iDMRs as hypermethylation of *m*ultiple *iD*MRs (HyMiD)-positive (55 tumors) (Fig. 1). We further defined tumors with hypermethylation in less than 10 iDMRs as HyMiD-negative (51 tumors). In addition, CpG island methylator phenotype (CIMP) is a well-known indicator of widespread CpG island promoter hypermethylation, first introduced by Toyota et al. [23]. Therefore, we analyzed CIMP status in all tumor tissues with five classical CIMP markers (*hMLH1*, *MINT1*, *MINT2*, *MINT31*, *p16*) using bisulfite pyrosequencing. We found that 25 tumors (24%) could be classified as CIMP-positive, which was defined as having at least two methylated markers among the five (Fig. 1). Comparison of HyMiD status with CIMP status revealed that HyMiD-positive status was correlated with CIMP-positive status (*p* = 0.005) (Table 1).

**Table 1:** Relationship between HyMiD and CIMP

	CIMP-positive	CIMP-negative	*p* value
HyMiD-positive	19	36	0.005
HyMiD-negative	6	45	

Fisher’s exact test

### Relationship between HyMiD status and *BRAF* or *KRAS* mutations

It was recently reported that the activated BRAF (V600E)-directed MAFG and activated KRAS (G13D)-directed ZNF304 pathways were responsible for CIMP in CRC [24, 25]. It was also reported that the *BRAF* and *KRAS* mutations were associated with CIMP-high and CIMP-low CRC, respectively [26–28]. The correlation between HyMiD-positive and CIMP-positive statuses therefore indicated the possibility of an association between HyMiD-positive status and the above two CIMP pathways. We explored the *BRAF* and *KRAS* mutations (codon 12, 13, 59, and 61) in tumor tissues. The *BRAF* mutation was identified in 5 tumors (4.7%), and *KRAS* mutations were identified in 42 tumors (39.6%) (Fig. 1 and Additional file 1: Table S2). CIMP-positive status was correlated with the *BRAF* mutation but not with *KRAS* mutations, including G13D (Additional file 1: Table S3). The lack of correlation between CIMP-positive status and *KRAS* mutations was an unanticipated result, but consistent with previous reports [29]. This discrepancy may be due to differing definitions of CIMP-positive and CIMP-high (as opposed to CIMP-low) [27, 28]. On the other hand, HyMiD-positive status was correlated with neither the *BRAF* mutation nor *KRAS* mutation (Table 2). Additionally, we investigated MAFG consensus binding sites in all iDMRs and found that few were present (Additional file 1: Table S4), supporting non-correlation of HyMiD-positive status with the *BRAF* mutation. These results suggest that the BRAF-directed MAFG and KRAS-directed ZNF304 pathways were not responsible for HyMiD-positive status in CRC.

**Table 2:** Relationship between HyMiD status and *BRAF* or *KRAS* mutation

	HyMiD-positive	HyMiD-negative	*p* value
*BRAF* mut (+)	2	3	0.464
*BRAF* mut (−)	53	48	
*KRAS* mut (+)	22	20	0.546
*KRAS* mut (−)	33	31	
*KRAS* G13D (+)	5	4	0.548
*KRAS* G13D (−)	50	47	

*BRAF* mut *BRAF* mutation (V600E), *KRAS* mut all *KRAS* mutations

Fisher’s exact test

### Relationship between HyMiD status and LINE-1 methylation

**Fig. 3:**
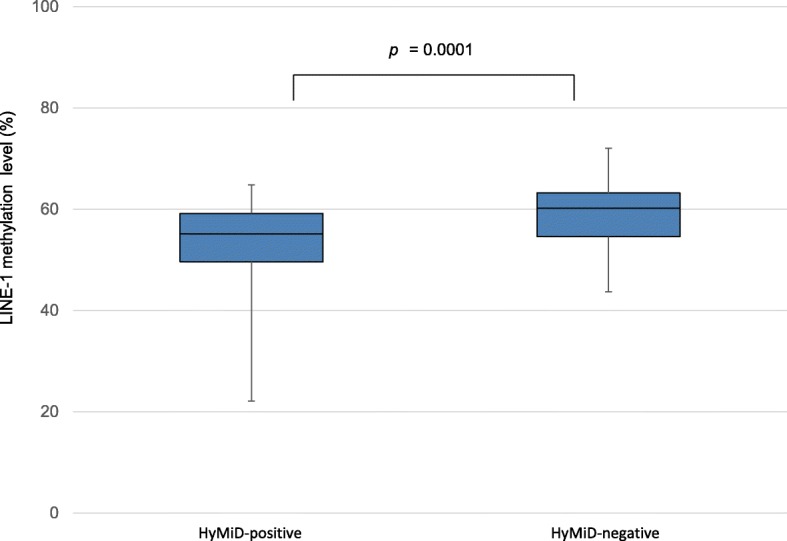
Inverse relationship between HyMiD status and LINE-1 methylation. LINE-1 methylation in HyMiD-positive tumors (median 55.1%, 22.1–64.8%) was significantly lower than in HyMiD-negative tumors (median 60.2%, 43.7–72.1%), indicating that an HyMiD-positive status was inversely correlated with LINE-1 methylation in CRC tissues. (*p* = 0.0001, Mann-Whitney *U* test)

The methylation level of LINE-1 retrotransposons, which constitute approximately 20% of the human genome, is a good surrogate marker of genome-wide methylation [30, 31]. LINE-1 methylation levels were investigated in tumor tissues by bisulfite pyrosequencing, and the relationship with HyMiD status was examined. The LINE-1 methylation level in HyMiD-positive tumors (median 55.1%, 22.1–64.8%) was significantly lower than in HyMiD-negative tumors (median 60.2%, 43.7–72.1%) (*p* = 0.0001), indicating an inverse relationship between HyMiD status and LINE-1 methylation (Fig. 3). LINE-1 methylation did not differ between CIMP-positive and CIMP-negative CRC (Additional file 1: Figure S1).

### Methylation status of iDMRs within the *IGF2/H19* domain

**Fig. 4:**
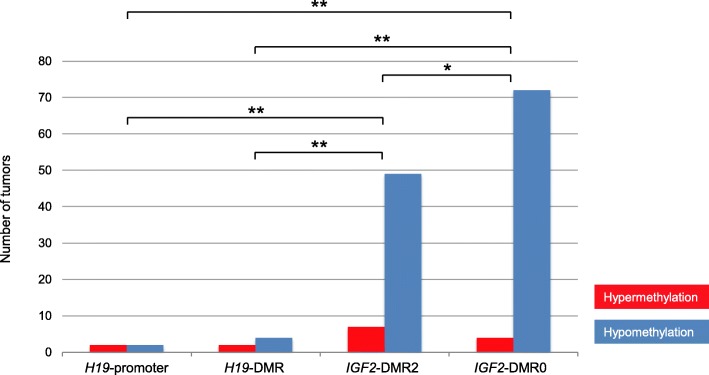
Aberrant methylation of four iDMRs within the *IGF2/H19* domain in tumors. Hypermethylation of the *H19*-promoter and *H19*-DMR were found in only a few samples. However, hypomethylation of *IGF2*-DMRs, especially DMR0, was comparatively overrepresented among iDMRs (*p = 0.0061, **p < 0.0001, *χ*^2^ test)

Among imprinting domains, the *IGF2/H19* domain has been particularly studied in CRC patients. Although four iDMRs (*H19*-DMR, *H19*-promoter, *IGF2*-DMR0, and *IGF2*-DMR2) are located within this domain, only a few studies have analyzed all of these iDMRs simultaneously. Therefore, we analyzed the methylation status of all iDMRs within the *IGF2/H19* domain. Methylation of *H19*-DMR and *H19*-promoter, which belong to the resistant iDMR group, was nearly unchanged (Figs. 1 and 4). Only two tumors were hypermethylated at both *H19*-DMR and *H19*-promoter. Four were hypomethylated at *H19*-DMR, and two were hypomethylated at *H19*-promoter. Conversely, hypomethylation at *IGF2*-DMR0 and *IGF2*-DMR2 was most frequently observed in 72 (67.9%) and 49 tumors (44.2%), respectively, as the two iDMRs belonged to the susceptible iDMR group (Figs. 1 and 4). Only four tumors were hypermethylated at *IGF2*-DMR0, and seven were hypermethylated at *IGF2*-DMR2. Hypomethylation of the *IGF2*-DMRs, especially DMR0, occurred at strikingly high frequency (Fig. 4).

### Allelic expression of *IGF2* and *H19*

We next analyzed the allelic expression of *IGF2* and *H19*. For this purpose, we searched for single nucleotide polymorphisms (SNPs) among the samples. We found that 39 and 40 patients were heterozygous for *IGF2* (rs680) and *H19* (rs2839702), respectively. Allelic expression was analyzed quantitatively in the heterozygous samples by pyrosequencing. There were four possible patterns of allelic *IGF2* expression in tumor tissue compared to matched adjacent normal mucosa: maintenance of imprinting (MOI) in both tissues, LOI in both tissues, LOI in tumor and MOI in normal mucosa, and MOI in tumor and LOI in normal mucosa. We found all patterns, and there was no significant difference in the number of sample pairs in each group (Additional file 1: Table S5). We further examined the relationship between *IGF2* LOI and *IGF2*-DMR0 hypomethylation in tumors and found no correlation (*p* = 0.901) (Table 3). Likewise, no correlation was found between *IGF2* LOI and *IGF2*-DMR2 hypomethylation (Additional file 1: Table S6). Thus, it is unlikely that hypomethylation of *IGF2*-DMRs caused *IGF2* LOI. As for *H19*, all tumor tissues except for one (ID 511) showed monoallelic expression, despite variation in iDMR methylation, strongly suggesting an absence of correlation between allelic expression of *H19* and aberrant methylation of iDMRs within the *IGF2*/*H19* domain.

**Table 3:** Relationship between *IGF2-*DMR0 hypomethylation and *IGF2* LOI in tumor

		Number of MOI cases	Number of LOI cases	*p* value
*IGF2-*DMR0 hypomethylation	(+)	9	21	0.901
	(−)	5	6	

*MOI* maintenance of imprinting, *LOI* loss of imprinting

Fisher’s exact test

### Relationship between aberrant methylation of iDMRs or *IGF2* LOI and prognosis

Since an association between *IGF2*-DMR0 hypomethylation and poor prognosis in CRC patients was previously reported [12], we also examined the relationship between clinicopathological factors predicting outcome and HyMiD status, *IGF2*-DMR0 hypomethylation, and *IGF2* LOI. However, we did not observe any correlations between the epigenetic features and clinicopathological factors (Additional file 1: Tables S7, S8, and S9).

**Fig. 5:**
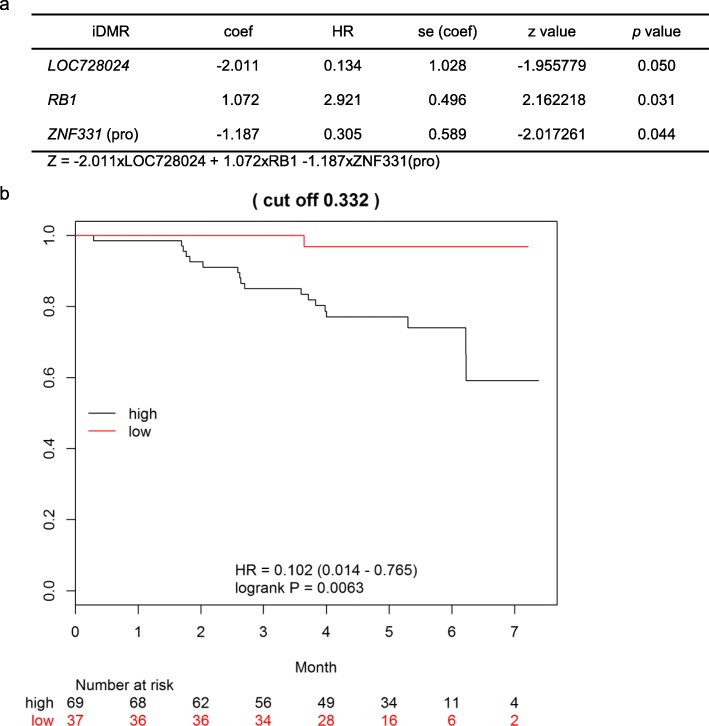
Cox proportional hazards regression analysis. **a** Model fitting summary. The prognosis score was computed as *Z* = − 2.011 × LOC728024 + 1.072 × RB1 – 1.187 × ZNF331-promoter. When aberrant methylation (hyper- or hypo-) at LOC728024, RB1, or ZNF331-promoter is found, the corresponding term is assigned a value of 1. coef: coefficient; se: standard error. z value = coef/se(coef). **b** Kaplan-Meier survival estimates. High prognostic scores (*Z* ≥ 0.332) were significantly associated with poor clinical prognosis compared low scores (*Z* < 0.332) (*p* = 0.0063, log-rank test, HR = 0.102 (95% CI 0.014–0.765))

We next analyzed the prognostic value of scores determined using a Cox proportional hazards regression model with stepwise selection. The prognosis score was computed as *Z* = − 2.011 × LOC728024 + 1.072 × RB1 – 1.187 × ZNF331-promoter. When aberrant methylation (hyper- or hypo-) at LOC728024, RB1, or ZNF331-promoter is found, the corresponding term is assigned a value of 1. The optimal cutoff value is 0.332, and HR = 0.102 (95% CI 0.014–0.765, *p* = 0.0063) between groups with *Z* ≥ 0.332 and *Z* < 0.332 (Fig. 5). The result of this analysis indicated that aberrant methylation of the three iDMRs was correlated with the patients’ prognosis and that the *Z* score was a potential prognostic marker.

## Discussion

In this study, we found that iDMRs were more susceptible to hypermethylation than hypomethylation in CRC tumor tissues and that the seven iDMRs were highly susceptible to aberrant hypermethylation. A recent report by Kim et al., using The Cancer Genome Atlas (TCGA) datasets from 22 cancer types, indicated that the direction of aberrant methylation is skewed towards hypermethylation and that hypermethylation of *PEG3*, *DLK1*, *PEG1*, and *GNAS* occurred more frequently [32]. The hypermethylated iDMRs identified by Kim et al. were ICRs, and almost entirely gametic iDMRs except for *DLK1*, whereas the hypermethylated iDMRs identified in the present study consisted of four gametic iDMRs and three somatic iDMRs. Furthermore, we could not find any consensus sequences among the five hypermethylated iDMRs. These results suggest that not only gametic DMRs but also somatic DMRs are aberrantly hypermethylated independent of genomic sequences in cancer, or at least in CRC. In addition, *PEG1* was a common iDMR in both studies, suggesting that *PEG1* hypermethylation is implicated in the development or progression of various cancer types.

HyMiD-positive status, which was defined as having at least ten hypermethylated iDMRs, was correlated with CIMP-positive status. Although the BRAF (V600E)-directed MAFG and KRAS (G13D)-directed ZNF304 pathways were responsible for CIMP in CRCs [24, 25], there was no correlation between HyMiD-positive status and mutations in *BRAF* or *KRAS*. While the number of tumors with *BRAF* mutation was low, the scarcity of MAFG-binding sites in iDMRs supported non-correlation between a HyMiD-positive status and *BRAF* mutation. These results suggest that the two pathways are not responsible for HyMiD-positive status in CRC, which may therefore be driven by other distinct molecular processes.

Genome-wide hypomethylation is believed to contribute to tumorigenesis by activating proto-oncogenes and inducing chromosomal instability (CIN) [33–35]. LINE-1 methylation is often used as a good surrogate marker for genome-wide methylation [31]. The results of analyses of the relationship between hypermethylation, such as CIMP, and genome-wide hypomethylation, such as LINE-1 hypomethylation, have been conflicting. CIMP-high tumors had higher LINE-1 methylation than non-CIMP-high tumors [36]; however, other studies including our present one showed that LINE-1 hypomethylation was independent from classical CIMP status [37, 38]. The discrepancy might be attributable to the array of CIMP markers and variations in the definition of CIMP. In this study, we identified a novel inverse association between HyMiD-positive with LINE-1 methylation. This difference in relationship to LINE-1 methylation between HyMiD and CIMP supports the existence of BRAF/KRAS-independent molecular processes in HyMiD.

The *IGF2/H19* domain has been well investigated because *IGF2* LOI is a common event in many cancers, including CRC. In this domain, *H19*-DMR plays a pivotal role in imprinting regulation in normal cells [2]. *H19*-DMR hypermethylation leads to *IGF2* LOI and *H19* silencing in Wilms tumor and hepatoblastoma [8, 9]. However, in this study, almost all tumors showed normal methylation of *H19*-DMR, strongly suggesting that the cause of *IGF2* LOI is not *H19*-DMR hypermethylation in CRCs. Some studies have proposed that *IGF2*-DMR0 hypomethylation could be the cause of *IGF2* LOI because of the association between *IGF2*-DMR0 hypomethylation and *IGF2* LOI [16, 39, 40]. However, another study contested this theory based on the lack of a tight link between *IGF2*-DMR0 hypomethylation and *IGF2* LOI, and it is improbable that the hypomethylation of paternally methylated *IGF2*-DMR0 could lead to reactivation of the silent maternal *IGF2* allele [18]. Because there was no correlation between *IGF2* LOI and *IGF2*-DMR0 hypomethylation, our results support the latter theory. We also could not find a correlation between *IGF2* LOI and *IGF2*-DMR2 hypomethylation. It is therefore clear that hypomethylation of *IGF2*-DMRs was not a cause of *IGF2* LOI. The cause of *IGF2* LOI in CRC might not be an alteration of DNA methylation within the *IGF2/H19* domain, but rather the dysfunction of any of the components related to imprinting regulation, such as the intrachromosomal loop, sequence alteration of *H19*-DMR, or expression of the CTCF or PRC2 complexes [41].

The association between *IGF2*-DMR0 hypomethylation and poor prognosis has been previously documented in CRC patients [12]. This raised the possibility that the methylation status of iDMRs and imprinting status might be associated with clinicopathological factors. Although we could not find any association between HyMiD, *IGF2*-DMR0 hypomethylation, or *IGF2* LOI and various clinicopathological factors, we were able to determine a prognostic score by including the status of aberrant methylation of the three iDMRs in a hazard regression model. This score would be useful to assess prognosis in CRC, although the mechanism for the role of aberrant methylation of iDMRs in mediating outcome is still unknown.

## Conclusions

We performed the first comprehensive methylation analysis of iDMRs in CRC patients. The iDMRs were more susceptible to hypermethylation than hypomethylation, but individual iDMRs varied in their susceptibility to aberrant methylation. We found that HyMiD-positive status was associated with CIMP-positive status and LINE-1 hypomethylation. We also confirmed that *IGF2*-DMR0 hypomethylation was not an appropriate surrogate marker for *IGF2* LOI. Moreover, we calculated a clinically relevant prognostic score based on aberrant methylation of the three iDMRs (LOC728024, RB1, and ZNF331-promoter). Further studies are required to understand the mechanisms behind aberrant methylation of iDMRs and *IGF2* LOI, and their significance in CRC patients.

## Methods

### Tissue samples

CRC patients with metastases were excluded from this study due to the very small number in this subgroup. Patients with preoperative chemotherapy or inflammatory bowel disease were also excluded to eliminate the influence of chemotherapy and chronic inflammation on DNA methylation [42–44]. Paired frozen tumor tissue and adjacent normal mucosa were obtained from 106 CRC patients who were admitted to Saga University Hospital. Dissected tissues were stored at − 80 °C followed by DNA and RNA extraction.

### DNA extraction and bisulfite conversion

Genomic DNA was extracted from each sample using the QIAamp DNA Mini Kit (Qiagen, Hilden, Germany) according to the manufacturer’s instructions. Five hundred nanograms of genomic DNA was subjected to bisulfite conversion using an EZ DNA Methylation Kit (Zymo Research, Irvine, CA, USA). The converted DNA was then eluted in 100 μl of water. The Human WGA Methylated & Non-Methylated DNA Sets (Zymo Research) were used as fully methylated and unmethylated DNA controls, respectively.

### Methylation analysis by bisulfite pyrosequencing

The methylation status of iDMRs, CIMP markers, and LINE-1 was analyzed by bisulfite pyrosequencing using a PyroMark Q24 pyrosequencing instrument (Qiagen) according to the manufacturer’s instructions. We designed primers for bisulfite-mediated PCR and pyrosequencing, using PyroMark Assay Design 2.0 software (Qiagen) in the majority of cases. The methylation percentage of each CpG site was calculated by PyroMark Q24 software (Qiagen). To validate the quantitative capability of bisulfite-pyrosequencing methylation analysis, all primer sets were evaluated using varying mixtures of the unmethylated control and the fully methylated control DNA: 0%, 25%, 50%, 75%, and 100% methylated DNA. All primers used in this study are indicated in Additional file 1: Table S10.

We compared the average methylation of CpG sites within each iDMR between tumor tissues and matched adjacent normal mucosa. Aberrant methylation of each iDMR was defined as the situation of tissue pairs in which the methylation difference between tumor and normal mucosa exceeded 15%. The methylation status of five classical CIMP markers (*hMLH1*, *MINT1*, *MINT2*, *MINT31*, *p16*) in tumor tissue was evaluated to determine CIMP status. Each marker was classified as positive (average methylation of CpG sites within a marker > 15%) or negative (< 15%). The tumor sample was classified as CIMP-positive if it was positive in two or more of the five markers and as CIMP-negative if it was positive in less than two markers. LINE-1 methylation level in tumor tissue was determined by the average methylation of CpG sites.

### *KRAS* and *BRAF* mutation analysis

Detection of the *BRAF* (V600E) mutation was performed by pyrosequencing, according to a previously published method [45]. A sample was considered mutation-positive if the mutant allele frequency was greater than 15%. Exons 2 and 3 of *KRAS* were amplified individually by PCR. The two PCR products were combined for each individual sample. Asymmetrical “Y-shaped” adaptors were ligated to PCR products after the end repair and A-tailing using KAPA Hyper Prep Kit (KAPA Biosystems, Wilmington, MA, USA). After adapter ligation, samples were amplified by PCR with indexed primers using the KAPA HiFi HotStart Ready Mix (KAPA Biosystems). The prepared amplicon libraries were sequenced using the MiSeq system (Illumina, San Diego, CA, USA). Mean read depth was > 1000 for all samples. A sample was considered mutation-positive if a mutation was present in at least 1% of the consensus reads [46].

### Allelic expression analysis of *IGF2* and *H19*

The genotyping of tumor tissue was performed by PCR-RFLP utilizing the two SNPs, *Hae*III (rs680) and *Alu*I (rs2839702) polymorphisms, located at *IGF2* exon 9 and at *H19* exon 5, respectively. In heterozygous samples, total RNA was extracted from both tumor tissue and matched adjacent normal mucosa using the ISOGEN II kit (Nippon Gene, Tokyo, Japan) following the manufacturer’s instructions. Total RNA was treated with RNase-free DNase I (Takara, Tokyo, Japan), and reverse transcription was performed using random primers and ReverTra Ace reagent mix (Toyobo, Osaka, Japan). To assess allelic expression of *IGF2* and *H19*, the RT-PCR products containing the SNPs were sequenced on a PyroMark Q24 pyrosequencing instrument (Qiagen), following the manufacturer’s instructions. When more than 85% of transcripts were recognized to be derived from a single allele, the expression mode was defined as monoallelic expression, or MOI. When less than 85% of transcripts were from one single allele, and more than 15% of transcripts were from another allele, the expression mode was defined as biallelic expression, or LOI.

### Statistical analysis

Correlations among HyMiD status, CIMP status, and *KRAS* and *BRAF* mutations were analyzed using Fisher’s exact test. Differences in LINE-1 methylation levels between HyMiD-positive and HyMiD-negative samples were analyzed using the Mann-Whitney U test. Frequencies of aberrant methylation of *H19*-promoter, *H19*-DMR, and *IGF2*-DMRs were analyzed with the *χ*^2^ test. Correlations between *IGF2* LOI and *IGF2*-DMR0 hypomethylation or *IGF2*-DMR2 hypomethylation were investigated by Fisher’s exact test. *p* values below 0.01 were considered to be statistically significant.

Correlations between clinicopathological factors and HyMiD status, *IGF2*-DMR0 hypomethylation, or *IGF2* LOI were statistically analyzed using the Kruskal-Wallis test, Fisher’s exact test, the Mann-Whitney U test, or the Log-rank test. In addition, all results were corrected for multiple hypotheses testing using the Bonferroni correction, and *p* values below 0.005 (0.05/10 = 0.005) were deemed statistically significant.

The prognosis score was computed based on a Cox proportional hazards regression model with stepwise selection. The optimal cutoff value for the score was computed by maximizing the log-rank test statistic. The Kaplan-Meier method was used to estimate survival distributions for each group. The log-rank test was used to compare groups, and the hazard ratio (HR) and 95% confidence interval (CI) were calculated. *p* values below 0.01 were considered statistically significant.

## Additional file

Additional file 1: **Table S1**. Information of patients analyzed in this study. Table S2 *KRAS* mutations identified in 106 CRC tissues. Table S3 Relationship between CIMP and *BRAF* or *KRAS* mutations. Table S4 MAFG consensus binding sites in the iDMRs. Table S5 *IGF2* imprinting status in paired tumors and normal mucosae. Table S6 Relationship between *IGF2*-DMR2 hypomethylation and *IGF2* LOI in tumors. Table S7 Relationship between clinicopathological factors and HyMiD status. Table S8 Relationship between clinicopathological factors and *IGF2*-DMR0 hypomethylation. Table S9 Relationship between clinicopathological factors and *IGF2* LOI. Table S10 Primers used in this study. Figure S1 Non-relationship between CIMP status and LINE-1 methylation. (PDF 478 kb)

## Abbreviations

CIMP: CpG island methylator phenotype
CIN: Chromosomal instability
CRC: Colorectal cancer
HyMiD: Hypermethylation of multiple iDMRs
iDMR: Imprinting-associated differentially methylated region
LINE-1: Long interspersed nuclear element-1
LOI: Loss of imprinting
MOI: Maintenance of imprinting
TCGA: The Cancer Genome Atlas
