# Supplementary material for: Comprehensive methylation analysis of imprinting-associated differentially methylated regions in colorectal cancer
Source: Clin Epigenetics. 2018 Dec 4;10:150. doi: 10.1186/s13148-018-0578-9 (PMC6278095; doi:10.1186/s13148-018-0578-9)
Supplement: Supplementary file 1 — Table S1. Information of patients analyzed in this study. Table S2 KRAS mutations identified in 106 CRC tissues. Table S3 Relationship between CIMP and BRAF or KRAS mutations. Table S4 MAFG consensus binding sites in the iDMRs. Table S5 IGF2 imprinting status in paired tumors and normal mucosae. Table S6 Relationship between IGF2-DMR2 hypomethylation and IGF2 LOI in tumors. Table S7 Relationship between clinicopathological factors and HyMiD status. Table S8 Relationship between clinicopathological factors and IGF2-DMR0 hypomethylation. Table S9 Relationship between clinicopathological factors and IGF2 LOI. Table S10 Primers used in this study. Figure S1 Non-relationship between CIMP status and LINE-1 methylation. (PDF 478 kb) [file 13148_2018_578_MOESM1_ESM.pdf]

Table S1. Information of patients analyzed in this study

		Right colon cancer	Left colon cancer	Rectal cancer	*p* value	Statistical method
Number of cases		35	36	35		
Age at diagnosis (median ± SD)		68 ± 9.9	69.5 ± 10.1	69 ± 9.2	0.902	Kruskal-Wallis test
Sex (Male : Female)		17:18	21:15	24 :11	0.237	χ^2 test
AJCC cancer stage	Ⅰ	9	6	8	0.762	
	Ⅱ	13	14	10		
	Ⅲ	13	16	17		
	Ⅳ	0	0	0		
Lymph node metastasis	(+)	13	16	17	0.620	
	(-)	22	20	18		
Pathological type	Differentiated	34	36	34	0.547	Fisher's exact test
	Poorly	1	0	1		

AJCC, American Joint Committee on Cancer

Table S2. ***KRAS*** **mutations identified in 106 CRC tissues.**

Mutations	Number of tumors	Frequency (%)	Patient ID
G12V	16	15.1%	322, 326, 336, 342, 343, 373, 380, 388, 413, 422, 426, 481, 505, 519, 521, 556
G12D	7	6.6%	275, 316, 361, 410, 444, 464, 558
G12S	3	2.8%	321, 330, 534
G12C	3	2.8%	285, 360, 428
G12A	1	0.9%	472
G13D	9	8.5%	313, 341, 374, 419, 456, 473, 478, 484, 557
G13C	1	0.9%	311
G13R	1	0.9%	425
A59E	1	0.9%	347

Table S3. Relationship between CIMP and *BRAF* or *KRAS* mutations.

	CIMP-positive	CIMP-negative	*p* value	Statistical method
BRAF mut (+)	5	0	0.001	Fisher's exact test
BRAF mut (-)	20	81		
KRAS mut (+)	11	31	0.387	
KRAS mut (-)	14	50		
KRAS G13D (+)	2	7	0.642	
KRAS G13D (-)	23	74		

BRAF mut: *BRAF* mutation (V600E), *KRAS* mut: all *KRAS* mutations

Table S4. MAFG consensus binding sites in the iDMRs.

DMR	Analyzed region[1]	Strand[2]	MAFG consensus binding site[3]	Number of consensus binding sites	Frequency of MAFG consensus binding site per 1 Kb
PPIEL	chr1: 40024626-40025540	-	non	0	0
ARHI-CG1<br>*ARHI*-CG2	chr1: 68515433-68517545	-	non	0	0
ARHI-CG3	chr1: 68512505-68513486	-	non	0	0
ZDBF2	chr2: 207114583-207136544	-1	ATAGCGCAGAATCTGCTGAAA	5	0.23
		-1	AATTTGCTTAGAGAGCATCAG		
		1	CTGATGCTCTCTAAGCAAATT		
		-1	AAAGTGCTGAGTATGTGTTAT		
		1	ATAACACATACTCAGCACTTT		
NAP1L5	chr4: 89618184-89619237	-	non	0	0
FAM50B	chr6: 3849082-3850359	-1	GATGCGCTGCTTCAGCACCTC	2	1.56
		1	GAGGTGCTGAAGCAGCGCATC		
ZAC	chr6: 144328078-144329888	-1	AGATTGCTTACTTAGCACATG	2	1.10
		1	CATGTGCTAAGTAAGCAATCT		
GRB10-DMR	chr7: 50848726-50851312	-	non	0	0
PEG10	chr7: 94285537-94287960	-	non	0	0
PEG1	chr7: 130130122-130134388	-1	CAAATGCTGATTTAGCACCTA	2	0.47
		1	TAGGTGCTAAATCAGCATTTG		
LOC728024	chr8: 37604992-37606088	-	non	0	0
TRAPPC9	chr8: 141108147-141111081	-	non	0	0
*H19*promoter<br>*H19*DMR	chr11:2018812-2024740	-	non	0	0
IGF2-DMR2	chr11: 2153991-2155112	-	non	0	0
IGF2-DMR0	chr11: 2168333-2169768	-	non	0	0
*Kv*DMR1	chr11: 2719948-2722259	-	non	0	0
RB1	chr13: 48892341-48895763	-	non	0	0
MEG3	chr14: 101290524-101293978	-	non	0	0
MEG8	chr14: 101370741-101371419	-	non	0	0
MAGEL2	chr15: 23892425-23894029	-	non	0	0
NDN	chr15: 23931451-23932759	-	non	0	0
SNRPN	chr15: 25200004-25201976	-1	CCTCTGCTGCGTCTGCGCAAC	2	1.01
		1	GTTGCGCAGACGCAGCAGAGG		
IGF1R	chr15: 99408496-99409650	-	non	0	0
ZNF597 (3')	chr16: 3481801-3482388	-	non	0	0
ZNF597 (TSS)	chr16: 3492828-3494463	-	non	0	0
ZNF331 (1)	chr19: 54040510-54042212	-	non	0	0
ZNF331 (2)	chr19: 54057086-54058425	-	non	0	0
PEG3	chr19: 57348493-57353271	-1	GGTTTGCCGAGTAGGCGCTGT	1	0.21
USP29	chr19: 57629548-57631498	-	non	0	0
L3MBTL	chr20: 42142365-42144040	-	non	0	0
NESP55	chr20: 57414039-57418612	-1	TTTGTGCTGGGTCATCAGAGC	2	0.44
		1	GCTCTGATGACCCAGCACAAA		
NESPAS-GNASXL	chr20: 57425649-57428033	-	non	0	0
GNAS1A	chr20: 57463265-57465201	-	non	0	0
GNASXL	chr20: 57428905-57431463	-	non	0	0
WRB	chr21: 40757510-40758276	-	non	0	0
NHP2L1	chr22: 42077774-42078873	-	non	0	0

[1]The analyzed regions were shown using the UCSC hg19 reference genome.

[2]Foward strand and reverse strand were indicated in 1 and -1, respectively.

[3]MAFG consensus binding site was identified by the JASPAR database. The relative profile score threshold was set at 80%.

Table S5. *IGF2* imprinting status in paired tumors and normal mucosae.

		Tumor			
		MOI	LOI	*p* value	Statistical method
Normal mucosa	MOI	4	3	0.225	Fisher's exact test
	LOI	10	22		

MOI, maintenance of imprinting; LOI, loss of imprinting

Table S6. Relationship between *IGF2*-DMR2 hypomethylation and *IGF2* LOI in tumors.

		Number of MOI cases	Number of LOI cases	*p* value	Statistical method
IGF2-DMR2 hypomethylation	(+)	5	14	0.326	χ^2 test
	(-)	9	13		

MOI, maintenance of imprinting; LOI, loss of imprinting

Table S7. Relationship between clinicopahtological factors and HyMiD status.

		HyMiD-positive	HyMiD-negative	*p* value	Statistical method
Number of cases		55	51		
Age at diagnosis (Median ± SD)		70.0 ± 10.0	68.0 ± 9.8	0.850	Kruskal-Wallis test
Sex	Male	27/55 (49.1%)	35/51 (68.6%)	0.032	Fisher's exact test
	Female	28/55 (50.9%)	16/51 (31.4%)		
Tumor location	Right	18/55 (32.7%)	17/51 (33.3%)	0.567	
	Left	21/55 (38.2%)	15/51 (29.4%)		
	Rectum	16/55 (29.1%)	19/51 (37.3%)		
Pathological type	Differentiated	54/55 (98.2%)	50/51 (98.0%)	0.733	
	Poorly	1/55 (1.8%)	1/51 (2.0%)		
Lymph node metastasis		22/55 (40.0%)	24/51 (47.1%)	0.296	
Lymphatic invasion		28/55 (50.9 %)	27/51 (52.9%)	0.494	
Vascular invasion		16/55 (29.1%)	16/51 (31.4%)	0.482	
AJCC cancer stage	I	17/55 (30.9%)	6/51 (11.8%)	0.053	
	II	16/55 (29.1%)	21/51 (41.2%)		
	III	22/55 (40.0%)	24/51 (47.0%)		
Tumor size (Median ± SD) (mm)		45.0 ±18.6	42.0 ± 16.0	0.556	Mann-Whitney U test
Survival rate		0.992	0.876	0.453	Log-rank test

AJCC, American Joint Committee on Cancer

Table S8. Relationship between clinicopathological factors and *IGF2- DMR0 hypomethylation.*

		IGF2-DMR0 hypomethylation (+)	*IGF2*-DMR0 hypomethylation (-)	*p* value	Statistical method
Number of cases		72	34		
Age at diagnosis (Median ± SD)		69.0 ± 10.2	69.5 ± 8.3	0.572	Kruskal-Wallis test
Sex	Male	42/72 (58.3%)	20/34 (58.8%)	0.566	Fisher's exact test
	Female	30/72 (41.7%)	14/34 (41.2%)		
Tumor location	Right	20/72 (27.8%)	15/34 (44.1%)	0.176	
	Left	28/72 (38.9%)	8/34 (23.5%)		
	Rectum	24/72 (33.3%)	11/34 (32.4%)		
Pathological type	Differentiated	71/72 (98.6%)	33/34 (97.1%)	0.541	
	Poorly	1/72 (1.4%)	1/34 (2.9%)		
Lymph node metastasis		35/72 (48.6%)	11/34 (32.4%)	0.085	
Lymphatic invasion		35/72 (48.6%)	20/34 (58.8%)	0.220	
Vascular invasion		20/72 (27.8%)	12/34 (35.3%)	0.285	
AJCC cancer stage	I	17/72 (23.6%)	6/34 (17.6%)	0.080	
	II	20/72 (27.8%)	17/34 (50.0%)		
	III	35/72 (48.6%)	11/34 (32.4%)		
Tumor size (Median ± SD) (mm)		45.0 ± 18.9	42.5 ± 13.8	0.753	Mann-Whitney U test
Survival rate		0.72	0.66	0.640	Log-rank test

AJCC, American Joint Committee on Cancer

Table S9. Relationship between clinicopathological factors and *IGF2* LOI.

		LOI	MOI	*p* value	Statistical method
Number of cases		25	14		
Age at diagnosis (Median ± SD)		69	69	0.510	Kruskal-Wallis test
Sex	Male	12 (48 %)	6 (42.9 %)	0.511	Fisher's exact test
	Female	13 (52 %)	8 (57.1 %)		
Tumor location	Right	8 (32 %)	5 (35.7 %)	0.458	
	Left	7 (28 %)	6 (42.9 %)		
	Rectum	10 (40 %)	3 (21.4 %)		
Pathological type	Differentiated	10 (40 %)	6 (42.9 %)	0.563	
	Poorly	15 (60 %)	8 (42.9 %)		
Lymph node metastasis		10 (40 %)	5 (35.7 %)	0.534	
Lymphatic invasion		12 (48 %)	8 (57.1 %)	0.416	
Vascular invasion		8 (32 %)	5 (35.7 %)	0.542	
AJCC cancer stage	I	6 (24 %)	3 (21.4 %)	0.915	
	II	9 (36 %)	6 (42.9 %)		
	III	10 (40 %)	5 (35.7 %)		
Tumor size (Median ± SD) (mm)		40 ± 16.9	46 ± 12.1	0.217	Mann-Whitney U test
Survival rate		0.74	0.92	0.218	Log-rank test

LOI: loss of imprinting, MOI: maintenance of imprinting, AJCC: American Joint Committee on Cancer

Table S10. Primers used in this study.

Analysis	Name of iDMR or gene	Primer name	Primer sequence	Location (GRCh37/hg19)	Total number of CpG sites	Number of analyzed CpG sites
Bisulfite-pyrosequencing for iDMRs		PPIEL F	Bio-GTTTAGGATGAGTAGGGTTATGGG			
	PPIEL	PPIEL R	ACCCCTTCTTTCCTTAATCT	Chr 1 : 40,024,626-40,025,540	5	5
		PPIEL S	CTAATACCCAATAAAATAAAACAC			
		ARHI-CG1 F	AACTCCCTCTAACTTCTTTCTCCCTTACCT			
	ARHI-CG1	ARHI-CG1 R	Bio-GTTTATAGGGTATTGTTGTTAAAATTTGT	Chr 1 : 68,510,575-68,510,716	4	4
		ARHI-CG1 S	CTTTCTCCCTTACCTAT			
		ARHI-CG2 F	TGGATTAGTTTTTAGATTGTTGTAGATGT			
	ARHI-CG2	ARHI-CG2 R	Bio-CCCCAAAAACTCACTCCTCC	Chr 1 : 68,511,601-68,511,797	3	3
		ARHI-CG2 S	GGTTAGTTTTTTATAGTTGGT			
		ARHI-CG3 F	AGTTTTGGTTTTAAGGAATAGAAGT			
	ARHI-CG3	ARHI-CG3 R	Bio-AACCCAACAACTAACAATAAATATTTTCA	Chr 1 : 68,515,139-68,515,364	5	5
		ARHI-CG3 S	AAGTTTTATAGGAAGATTAGAG			
		ZDBF2 F	GGTTTTAAATGATTTTGGGTAGTTTG			
	ZDBF2	ZDBF2 R	Bio-CCCCTAATAACTTTATCTAACTTCTCT	Chr 2 : 207,125,719-207,125,922	3	3
		ZDBF2 S	GATTTTGGGTAGTTTGG			
		NAP1L5 F	AGGGTAGTAATAGGAGGAATTTGGTGTAGT			
	NAP1L5	NAP1L5 R	Bio-AAAACCCAACTCAAAACCTAAAAAAC	Chr 4 : 89,618,184-89,619,237	4	4
		NAP1L5 S	GGGAGTTTTTTTAGATTT			
		FAM50B F	GGGTTTTGAGGAGAGTGTTAGG			
	FAM50B	FAM50B R	Bio-ACCAAAAACCCCTCCTCCAAAATC	Chr 6 : 3,849,403-3,849,577	3	3
		FAM50B S	GGAAATAGGAAGTGGAG			
		ZAC F	ACCTTAACTTTACCCCCCAC			
	ZAC	ZAC R	Bio-GGTTGAATGATAAATGGTAGATGT	Chr 6 : 144,317,462-144,317,725	7	7
		ZAC S	AACCTCCTCCTACCA			
		GRB10 F	GTTAGGTTTTGGAGTATAATAGGAATT			
	GRB10-DMR	GRB10 R	Bio-AATTACCATAAAAACCAAAAATCC	Chr 7 : 50,848,726-50,851,312	6	6
		GRB10 S	GAATTTTAGGATTAAATTTATGTGA			
		PEG10 F	TTGGTTTAGGTGTGGGATTTT			
	PEG10	PEG10 R	Bio-AAACATTCTAAAATACTACTCCATCTC	Chr 7 : 94,285,537-94,287,960	5	5
		PEG10 S	AGGTGTGGGATTTTATTT			
		PEG1 F	GTGGTTAGGAGTTTGGTATGTTGA			
	PEG1 (*MEST*)	PEG1 R	Bio-AAACCCACCACCAAACTAAT	Chr 7 : 130,130,122-130,134,388	6	5
		PEG1 S	GTTGTTGTAAAGGAAATTT			
		LOC728024 F	GGTATGGTTTTTAGGAGTAATTTGA			
	LOC728024	LOC728024 R	Bio-AACCTATAACCCTAAACTAATAACTT	Chr 8 : 37,604,992-37,606,088	8	7
		LOC728024 S	GAAAAATTTTAAGTAAAGAAGAGGG			
		TRAPPC9 F	Bio-TTTTGTTGGTAGGGTTGTGT			
	TRAPPC9	TRAPPC9 R	TCATCCTCAAAACCCTATCACC	Chr 8 : 141,108,147-141,111,081	4	4
		TRAPPC9 S	AAACCTAAATACAAAATCC			
		H19-Promoter F	GTTTGGGAGAGTTTGTGAGG			
	H19-promoter	H19-Promoter R	Bio-CCCCAAACCCATTCCCATCCAATTAAC	Chr 11 : 2,015,729-2,015,932	4	4
		H19-Promoter S	GTTTATAGTTTGTTAGTAGAGTG			
		H19DMR F	TGGGTATTTTTGGAGGTTTTTTT			
	H19-DMR	H19DMR R	Bio-ATAAATATCCTATTCCCAAATAA	Chr 11 : 2,019,871-2,024,740	4	4
		H19DMR S	GTTTYGGGTTATTTAAGTTA			
		IGF2-DMR2 F	Bio-GGTTTTGGGTGGGTAGAGT			
	IGF2-DMR2	IGF2-DMR2 R	CCCCAAATACCCCCTAAACAAATTCTTC	Chr 11 : 2,153,991-2,155,112	5	5
		IGF2-DMR2 S	CCTACCTACCCTCCT			
		IGF2-DMR0 F	TGAGGATGGGTTTTTGTTTGGTAT			
	IGF2-DMR0	IGF2-DMR0 R	Bio-TCCTCAATCCACCCAAAATAATAT	Chr 11 : 2,168,333-2,169,768	3	3
		IGF2-DMR0 S	AAAAGTTATTGGATATATAGT			
		KvDMR1 F	AGGGAAGTTTTAGGGTGTGAATTTTTAGAG			
	*Kv*DMR1	KvDMR1 R	Bio-CCAAACCACCCACCTAACAAAAAAC	Chr 11 : 2,719,948-2,722,259	6	6
		KvDMR1 S	TGGTAATGTTTGGTATTT			
		RB1 F	GGTAGGGTAGTTTTGGAAATGTTTAAG			
	RB1	RB1 R	Bio-AACCACAAACCCTTACCC	Chr 13 : 48,892,341-48,895,763	6	6
		RB1 S	AGTTTTGGAAATGTTTAAGAT			
		MEG3-CG7 F	GTTAGGTAGGGTGAATTTAGGTATAATGTGTG			
	MEG3	MEG3-CG7 R	Bio-AACAACCTCTAACTTACATCTAAAAACCAATT	Chr 14 : 101,290,524-101,293,978	7	7
		MEG3-CG7 S	ATGGTTAAGGTGGGGTTG			
		MEG8 F	GAGGGGGATAGTTAAGAGGGA			
	MEG8	MEG8 R	Bio-ACAACTCCCCCAACCTACTACATTACA	Chr 14 : 101,370,741-101,371,419	7	7
		MEG8 S	TTGGTAGAGGAGTTTTTAATTG			
		MAGEL2 F	GTTAATTGGAGGTGGATTTTAAGG			
	MAGEL2	MAGEL2 R	Bio-AAACAACCACCTAAACATTCTCTTCTCTC	Chr 15 : 23,892,425-23,894,029	5	5
		MAGEL2 S	GAATAGTAGGGAGGTTATAGATAGG			
		NDN F	GTAGGGTTTTTGAGTTTAGGAGTTTTT			
	NDN	NDN R	Bio-AATCCCTCTCCCAACAAATAAAACCTC	Chr 15 : 23,931,451-23,932,759	7	6
		NDN S	GATTTTTGGAGAGGAAG			
		SNRPN F	GGGAGGGAGTTGGGATTTTTG			
	SNRPN	SNRPN R	Bio-AAACCACCCACACAACTAACCTTACCC	Chr 15 : 25,200,004-25,201,976	7	7
		SNRPN S	AGTTGGGATTTTTGTATTG			
		IGF1R F	Bio-TAAGGAGGGGTTGGTTGGAG			
	IGF1R	IGF1R R	AACCCCACTCCCTTTTCTAT	Chr 15 : 99,408,496-99,409,650	6	6
		IGF1R S	CCCAACCTAACCTAAAACCAC			
		ZNF597(2) F	ATGTAGTGGATATGGTTAGGTATATG			

	ZNF597 (3')	ZNF597(2) R	Bio-ATCTCCAACTTAACCACCAACTA	Chr 16 : 3,481,801-3,482,388	5	5
		ZNF597(2) S	ATAGTTTATTTGTTTTAGGAATG			
	ZNF597 (TSS)	ZNF597 F	GGAGGGGGTTAGGTAGAG	Chr 16 : 3,492,828-3,494,463	5	5
		ZNF597 R	Bio-ATCCCTTTAAAAACTATTCTCTCC			
		ZNF597 S	AGGTATTTAGATTTATTTGGGTT			
	ZNF331 (pro)	ZNF331 F	Bio-AGGGATTAGGAAGGGTTTAGT	Chr 19 : 54,040,510-54,042,212	5	5
		ZNF331 R	CCCAAACCCCCTATTCCAACATACAAC			
		ZNF331 S	CCCAAACAAAAACCTC			
	ZNF331 (int)	ZNF331(2) F	GTTGGGTGAGGGAGGTTTT	Chr 19 : 54,057,086-54,058,425	5	5
		ZNF331(2) R	Bio-CCCCATCACCCCCAATAA			
		ZNF331(2) S	GGTGAGGGAGGTTTTTT			
	PEG3	PEG3 F	GGTGGTTTTAGGTTAGTTAGAAAGG	Chr 19 : 57,348,493-57,353,271	5	5
		PEG3 R	Bio-ACCAAACTATAAATAATTAATACCTTTT			
		PEG3 S	AGATTTTGTAGTAGTTTTTAGATT			
	USP29	USP29 F	TTTTTAGAGGGAGAGATGGAAGTTGG	Chr 19 : 57,629,548-57,631,498	6	6
		USP29 R	Bio-ACCACAAACCCCCAAATCTTAAAAAA			
		USP29 S	TGATTGTATTTGGGTTT			
	L3MBTL	L3MBTL-CG3 F	TATGAGGAGAAGAGAGGGTTATGGTAT	Chr 20 : 42,142,365-42,144,040	5	5
		L3MBTL-CG3 R	Bio-AAAACCCAACTCAAAACCTAAAAAAC			
		L3MBTL-CG3 S	GGGAGTAGTAATTTTTAGATTTTTT			
	NESP55	NESP55 F	TTTTTTTTGGGGTTTTGGGGTTTTT	Chr 20 : 57,414,039-57,418,612	7	7
		NESP55 R	Bio-AACAAACCTTAAACTATCCCTCCCC			
		NESP55 S	TGGAGTGTTTGGGTATTA			
	NESPAS-GNASXL	NESPAS-GNASXL F	GGATATGGGTGGGAGGTTTAA	Chr 20 : 57,425,649-57,428,033	5	5
		NESPAS-GNASXL R	Bio-CCCTAAAACCCCACCTCTC			
		NESPAS-GNASXL S	GGGTGGGAGGTTTAATA			
	GNAS1A	GNAS 1A F	TGTTGAAGATGGTTATGAAGTTTAAAGTT	Chr 20 : 57,463,265-57,465,201	8	8
		GNAS 1A R	Bio-TATCCCAATCAACCCAAACCCTACTC			
		GNAS 1A S	AAGTTTAGGAGGAGGTT			
	GNASXL	GNASXL F	ATGAAGGGGTGGTTAGTAG	Chr 20 : 57,428,905-57,431,463	6	6
		GNASXL R	Bio-CTCCCCCAAAAATACCTTCTTAACCTT			
		GNASXL S	GGATGTTTTAGTTGGTTTTAGTA			
	WRB	WRB F	TTGGAGGGGGTTGAGGGTTTA	Chr 21 : 40,757,510-40,758,276	5	5
		WRB R	Bio-TACCAATCCTAAAACACCTATACAATAC			
		WRB S	GGGTTGAGGGTTTAT			
	NHP2L1	NHP2L1 F	Bio-GGATGTTTAGTTTTTAAGAGTAGGAAGTG	Chr 22 : 42,077,774-42,078,873	4	4
		NHP2L1 R	CCCAACCCCCTTCCTTTAA			
		NHP2L1 S	CCTAATATACCAACTAACCCTAA			
Bisulfite-pyrosequencing for CIMP markers	*hMLH1*	hMLH1 F	TTGATTGGTATTTAAGTTGTTTAATT	Chr 3 : 37,034,823-37,107,380	3	3
		hMLH1 R	Bio-CCAATTCTCAATCATCTCTTTAATA			
		hMLH1 S	AGTTATAGTTGAAGGAAGAA			
	MINT1	MINT1 F	GGTTTTTTGTTAGYGTTTGTATTT	Chr 5 : 75,378,997-75,649,764	3	3
		MINT1 R	Bio-ATTAATCCCTCTCCCCTCTAAACTT			
		MINT1 S	TTTAGTAAAAATTTTTTGGG			
	MINT2	MINT2 F	AGTGTTAGAAAAATGTGTTG	Chr 2 : 58,654,934-59,290,901	3	3
		MINT2 R	Bio-CTACAATTAAACATCAATTATAT			
		MINT2 S	GAATTTTAGTATTTAAGTT			
	MINT31	MINT31 F	GTTTAGGGGTGATGGTTTTAGT	Chr 17 : 48,633,568-48,639,469	4	4
		MINT31 R	Bio-AAAACACTTCCCCAACATC			
		MINT31 S	GGTGATGGAGGTTAT			
	p16	p16 F	GGTTGTTTTYGGTTGGTGTTTT	Chr 9 : 21,967,751-21,995,300	4	4
		p16 R	Bio-ACCCTATCCCTCAAATCCTCTAAAA			
		p16 S	GGAGTTAATAGTATTTTT			
Bisulfite-pyrosequencing for LINE-1 methylation	LINE-1	LINE-1 F	TTTTGAGTTAGGTGTGGGATATA		4	4
		LINE-1 R	Bio-AAATCAAAAAATTCCCTTTC			
		LINE-1 S	AGTTAGGTGTGGGATATAGT			
KRAS mutation analysis	*KRAS* exon2	KRAS F1	GGTGAGTTTGTATTAAAAGGTACTGG			
		KRAS R1	CCTTTATCTGTATCAAAGAATGGTC			
	KRAS exon3	KRAS F2	CCAGACTGTGTTTCTCCCTTC			
		KRAS R2	GCATGGCATTAGCAAAGACTC			
Pyrosequencing for BRAF (V600E) mutation analysis	*BRAF*	BRAF F	ATGCTTGCTCTGATAGGAA			
		BRAF R	Bio-GCATCTCAGGGCCAAA			
		BRAF S	GGTGATTTTGGTCTAGCTAC			
Genotyping of *IGF2* (rs680)	*IGF2*	IGF2 P2-2	GGACTTTGAGTCAAATTGGC			
		IGF2 P5-2	GGGTTGTTGCTATTTTCGGA			
Genotyping of *H19* (rs2839702)	*H19*	H19 331	CGGACACAAAACCCTCTAGCTTGGAAATGA			
		H19 332	AATGGAATGCTTGAAGGCTGCTCCGT			
Pyrosequencing for allelic-specific expression analysis	*IGF2*	IGF2-allele F	Bio-GGACTTTGAGTCAAATTGGC			
		IGF2-allele R	GGGTTGTTGCTATTTTCGGA			
		IGF2-allele S	GTGCCCACCTGTGATTTCTG			
	H19	H19-allele F	Bio-CGGACACAAAACCCTCTAGCTTGGAAATGA			
		H19-allelle R	GCGCTGCTGTTCCGATGGTG			
		H19-allele S	GGCTGATGAGGTCTGGTTCC			

F: forward primer, R: reverse primer, S: sequence primer, Bio: biotinylated primer

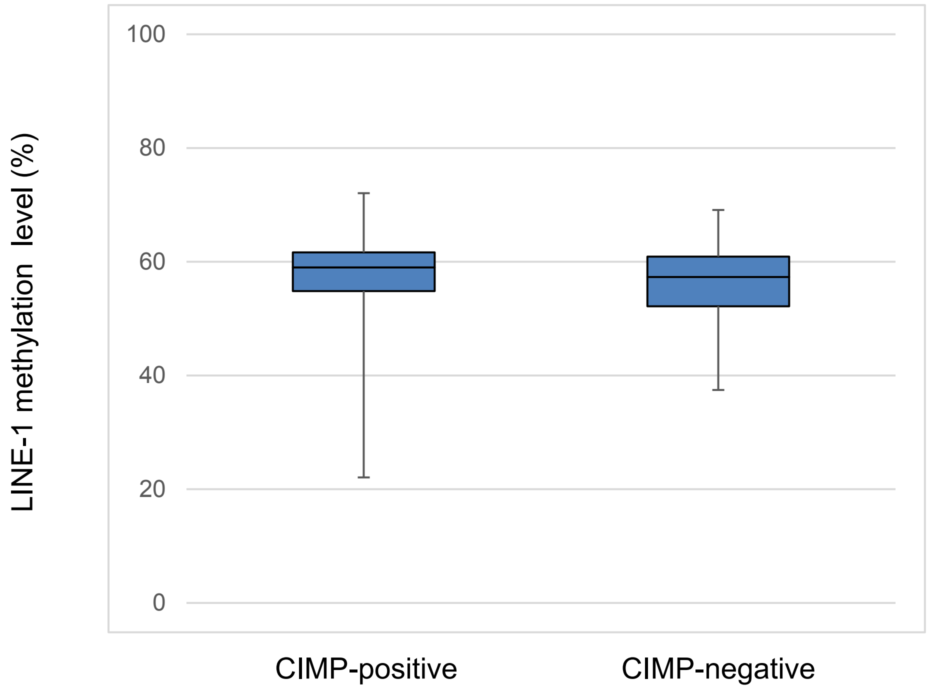

Figure S1. Non-relationship between CIMP status and LINE-1 methylation.

LINE-1 methylation in CIMP-positive tumors (median: 59.0%, ranging from 22.1 to 72.1%) was not significantly different from that in CIMP-negative tumors (median: 57.3%, ranging from 37.5 to 69.1%) (p = 0.235, Mann-Whitney U test).
